# Thiacloprid impairs reproductive functions of male Wistar rats

**DOI:** 10.1007/s00210-024-03025-7

**Published:** 2024-03-05

**Authors:** Aya Abdel Nasser Mahmoud, Ebtehal Altohamy Ahmed, Amel Ramadan Omar

**Affiliations:** Zoology Department, Faculty of Science, Cairo University, Giza, Egypt

**Keywords:** Male infertility, Neonicotinoids, Reproductive toxicity, Antioxidant enzymes, Comet assay

## Abstract

Global male infertility correlated to the rise of endocrine-disrupting chemicals, including insecticides, has grown into a pressing problem. Thiacloprid is one of the most commonly used neonicotinoids that accounts for more than 25% of the global pesticide industry. However, its impact on the reproductive system and male fertility has not been fully elucidated. The object of this study was to explore the adverse effects of thiacloprid on male Wistar rats’ reproductive system. Thirty healthy male rats were separated into one of three groups: control group, and two groups that were orally administered with low (22.5 mg/kg) and high dose (62.1 mg/kg) of thiacloprid for 56 days. Thiacloprid significantly (*p*<0.05) reduced body weight and relative testicular weight, as well as sperm quality (count, motility, viability, and morphology), in a dose-dependent manner. THIA-treated groups revealed a large effect (*d* > 0.8) on semen quality with Cohen’s *d* of (6.57, 8.82), (20.14, 23.54), and (2.81, 9.10) for count, motility, and viability respectively. Meanwhile, the serum testosterone level dropped while the levels of luteinizing and follicle-stimulating hormones increased. 17ꞵ-hydroxy steroid dehydrogenase and 3ꞵ-hydroxy steroid dehydrogenase levels were significantly decreased in a dose-dependent manner. The activity of the tested antioxidant enzymes catalase (CAT), glutathione reduced (GSH), and superoxide dismutase (SOD) exhibited a considerable decrease compared to the control group with a significant elevation in the lipid peroxidation activity as indicated by malondialdehyde (MDA) level. The testicular histology revealed degenerative changes in spermatogenic cells and interstitial tissue. Comet assay revealed DNA fragmentation in treated groups’ testicular tissue. Thiacloprid exposure interferes with reproductive function and impairs male Wistar rat fertility. Such harmful consequences may also develop in humans frequently exposed to thiacloprid.

## Introduction

Male infertility became a growing public health concern worldwide, accounting for about 50% of all infertility instances. Despite the association of most male infertility cases to decreased sperm quality **(**Levine et al. 2017), 25% of infertile men are idiopathic (Kumar and Singh 2015). Several studies have emphasized that occupational or environmental exposure to hazardous chemicals, including insecticides, induced male infertility, and the potential changes in testicular function (Pineau 2020; Agarwal et al. 2021; Selvaraju et al. 2021). The ongoing use of pesticides contaminates the environment and eventually makes its way into human life risking their health by chronic diseases including cancer, neurological diseases, diabetes, and multiple sclerosis (Gangemi et al. 2016; Saravi and Dehpour 2016). Furthermore, pesticides have received the most attention among environmental toxicants for their potential harmful effects on male reproduction (Perry 2008; La Rocca et al. 2015; Kong et al. 2017; El namaky et al. 2018; Moreira et al. 2021).

Many studies have established a correlation between the exposure to pesticides and the occurrence of reproductive problems through interfering with neurotransmission and endocrine regulation, resulting in changes in reproductive organ physiology and spermatogenesis disturbance (Perry et al. 2007; Martenies and Perry 2013; Neto et al. 2016; Tavares et al. 2016). These contaminants act as endocrine disruptors (EDCs), mimicking natural endogenous hormones and interfering with their action in the endocrine system, altering sexual identity, fertility, or behavior (Roy et al. 2017; Rodprasert et al. 2019).

The neonicotinoids class of neurotoxic pesticides is the most widely used type of systemic insecticide for controlling insect pests on crops and pets on a global scale, accounting for almost 25% of the worldwide pesticide industry (Craddock et al. 2019). Neonicotinoids exhibit exceptional potency and safety due to insects’ greater selectivity for nicotinic acetylcholine receptors (nAChRs) than mammals (Han et al. 2018). Nonetheless, because of their persistence and water solubility, their residues remain in the environment and may endanger human and animal health (Chen et al. 2020; Li and Kannan 2020; Wang et al. 2020; Mahai et al. 2021). Some animal studies have found that these insecticides can harm mammalian reproductive systems, such as delayed testicular development, spermatogenesis damage, and lower sperm quality, despite their declared low risk to mammalian cells and organs (Bal et al. 2012a, b; Bal et al. 2013; Caron-Beaudoin et al. 2016, 2017).

One of the neonics is thiacloprid (THIA) was found to pose a risk to non-target species. Previous studies on rats and zebrafish marked the liver as the primary THIA target organ (Alarcan et al. 2020; Abou-Zeid et al. 2021; Xie et al. 2022). Other accumulated evidence from studies on rats, mice, rabbits, and chick embryos suggested that it is also nephrotoxic (Kammoun et al. 2019), embryotoxic (EPA 2013; Babeľová et al. 2017), and neurotoxic with consequences for brain development (Farag et al. 2021; Farag et al. 2022). Prior research has identified alterations in honeybees’ immune response (Brandt et al. 2016) and behavior (Tison et al. 2016) following treatment of pure thiacloprid. Thiacloprid was also linked to prostate toxicity in rats, mice, and dogs (EPA 2013; EFSA 2019).

Thiacloprid has been demonstrated to dramatically influence antioxidant enzymes such as superoxide dismutase (SOD) and catalase (CAT), along with non-enzymatic antioxidants like glutathione (GSH). On the other hand, an elevation in malondialdehyde (MDA) and nitrous oxide (NO) in the thymus, liver, and spleen of rats (Abou-Zeid et al. 2021). Likewise, THIA induced oxidative stress in carp and zebrafish (Velisek and Stara 2018; Wang et al. 2020) and chick (Farag et al. 2023). The maintenance of fertility and regulation of spermatogenesis are contingent upon the balance of sex hormones and the level of oxidative stress (Kegley et al. 2010).

The male reproductive system development was influenced by the continuous administration of THIA at dosages of 50 and 100 mg/kg during puberty. Exposure to THIA resulted in a decrease in the mRNA levels of genes involved to spermatogenesis and an increase in the rate of sperm abnormalities. Furthermore, THIA decreased the concentration of testosterone and FSH in the bloodstream and suppressed the activity of genes and proteins involved in the production of testosterone (Zou et al. 2023). Also, thiacloprid, combined with five other pesticides, induces endocrine disruption in female mice. This disruption occurs through the disturbance of folliculogenesis, particularly in the luteinization process, a highly sensitive and critical phase in female reproduction (Dopavogui et al. 2022).

Reactive oxygen species (ROS), commonly referred to as free radicals, have the ability to infiltrate DNA, resulting DNA damage or mutation (Chang et al. 2009; Pouech et al. 2012). Moreover, high levels of ROS in the testes were discovered to worsen Leydig cell harms and lower the levels of plasma testosterone in adult male rats (Bal et al. 2012b).

Hartman et al. (2021) reported that thiacloprid exposure during pregnancy might negatively affect male spermatogenesis by interfering with epigenetic systems governing meiosis. DNA damage and apoptosis were found to have been caused by THIA exposure in both bovine lymphocytes (Schwarzbacherová et al. 2019) and human hepatocellular carcinoma cells (Senyildiz et al. 2018).

The insecticide exposure duration and timing play a significant role in the severity of fertility disruption (Fucic et al. 2021), and there is no satisfied data regarding reproductive toxicity of long-term exposure to thiacloprid. This study aims to explore the effect of thiacloprid exposure for 8 weeks on the male reproductive system of Wistar rats, as well as the implications for male fertility in terms of semen quality, hormonal disruptions, testicular enzymes activity, oxidative stress, histopathological changes, and DNA damage.

## Materials and methods

### Thiacloprid

The chemical used in this study, thiacloprid, is one of the neonicotinoid insecticides with the chemical formula N-{3-[(6-chloro-3-pyridinyl) methyl]-1,3-thiazolan-2-yliden} cyanamide. It was purchased from Starchem, Egypt, under the commercial name Blanch (48% SC) water-based suspension concentrate at 480 g/l. The daily administration of the solution involved the preparation of a fresh batch, which was then modified on a weekly basis to account for variations in body weight.

### Experimental animals

Healthy adult male Wistar rats, aged 8 weeks (weighing 170 ± 20g), were procured from the National Research Center (Egypt). Animals were housed for 1 week as acclimatization time in polycarbonate cages. During this time, the animals had unrestricted access to both food and water. This acclimatization period took place prior to the commencement of the experiment. The rats were kept in a controlled 12-h dark/light cycle with a temperature (23±1°C) and a relative humidity (55±10%). Animals were used in experiments in accordance with the National Institutes of Health (NIH) animal care and handling guidelines. The Institutional Animal Care and Use Committee (IACUC), Faculty of Science, Cairo University, approved this study (approval number CU/I/F/16/22).

### Experimental design

Thirty male rats were distributed into three groups (ten per group). They were treated daily by oral gavage for 56 days, corresponding to the time of complete spermatogenesis (Creasy 1997); the present study was designed as an exploratory study to investigate the reproductive toxicity of THIA as follows.

- **Group I**: (the control group) was administrated distilled water as a vehicle by gastric gavage.
- **Group II**: (Low dose) Rats received 22.5 mg/kg of thiacloprid, according to Kammoun et al. (2017).
- **Group III**: (High dose) Rats received 62.1 mg/kg equivalent to 1/10 LD_50_ of thiacloprid, according to Abou-Zeid et al. (2021).

After the experiment period, the weights of rats were recorded and anesthetized with 50 mg/kg of sodium pentobarbital intraperitoneally (Abd El-Rahman and Omar 2022). Serum samples were separated from the retro-orbital plexus blood by centrifugation for 15 min at 3000 rpm (Mehanna et al. 2021). Subsequently, the sera were kept at −20 °C for hormonal analysis. Testes, epididymis, and seminal vesicles were harvested, detached, and cleared from the surrounding tissues. These tissues were washed in normal saline and weighed. The left testis was put in 10% buffered formalin for histopathological analysis, while the right testis was stored at −20 °C for lipid peroxidation, enzymatic antioxidant, and comet assay.

### Semen collection and analysis

The collection of epididymal sperm was achieved through the fragmentation of the cauda epididymis into smaller segments within 1 ml of normal saline in a warm petri dish at 37 °C for 5 min to allow spermatozoa to disperse into the medium (Halvaei et al. 2012). Epididymal tissue was removed from the suspension and discarded. Then, the suspended sperm of each rat was collected for semen analysis.

#### Sperm motility and count

A sperm count was performed immediately after liberation from the cauda epididymis, and at the same time, the motility was assessed. The sperm count was conducted by dropping 10 μl of diluted sperm onto a pre-warmed Neubauer hemacytometer slide and allowing it to settle for 5 min before microscopic analysis. The resulting sperm count was calculated in a million/ml suspension. At least 200 sperm were counted in ten fields while 100 sperm were examined to evaluate sperm motility. Sperm samples were examined under a light microscope at 400× magnification, according to Halvaei et al. (2012).

#### Sperm viability and morphology

The eosin-nigrosin staining test was applied to assess sperm morphology and viability. Semen samples were mixed with stain (1% eosin–10% nigrosin). A droplet measuring 15μl of this mixture was carefully deposited onto a glass slide. The specimen was meticulously prepped and afterwards subjected to air-drying under ambient conditions. These slides were inspected at 400× magnification by a light microscope. The alive spermatozoa that were exhibited no staining, appearing white, whereas the deceased cells were stained red. The percentage of alive and dead spermatozoa and morphological abnormalities was calculated by enumeration of a minimum 200 spermatozoa (Halvaei et al. 2012).

### Reproductive hormones and analysis

Testosterone (Cusabio, USA, Catalog number. CSB-E05097r), luteinizing hormone (LH) (Novus Biologicals, USA, Catalog number. NBP2-61257), follicle-stimulating hormone (FSH) (Abnova, Taiwan, Catalog number. KA2330) levels were detected using enzyme linked immunosorbent assay (ELISA) kits, following the manufacturer’s guidelines.

### Testicular steroidogenic enzyme activities

Testicular enzymes, including 3-beta-hydroxysteroid dehydrogenase (3ꞵ-HSD) and 17-beta-hydroxysteroid dehydrogenase (17ꞵ-HSD) were detected using ELISA kits (Fine Biotech Co., China, Catalogue No.: ER0665 and Biomatik, Canada, Catalogue No.: EKU10595 respectively), according to the manufacturer’s directives.

### Evaluation of oxidative stress markers

Frozen testicular tissues were homogenized in PBS (1:10 ml) and the homogenates were centrifuged at 12,000 rpm for 20 min. The supernatant was utilized to evaluate oxidative biomarkers. Assay kits were purchased from Biodiagnostic, Egypt, were utilized to assess malondialdehyde (MDA), catalase (CAT), reduced glutathione (GSH), and superoxide dismutase (SOD), and as indicated by the manufacturers with catalogue numbers MD2529, CA2517, GR2511, and SD2521 respectively. The measurement of testicular activity for MDA, CAT, GSH, and SOD was conducted using a UV-2100 spectrophotometer (Qualitest, USA) based on the available evidence of Ohkawa et al. (1979), Aebi (1984), Beutler et al. (1963), and Nishikimi et al. (1972), respectively.

### Histopathological studies

The left testis of every rat was isolated and fixed in 10% neutral buffered formalin. Subsequently, it underwent a process of dehydration using a variety of ethanol concentrations, followed by clearing in xylene, and finally embedding in paraffin. The tissues were prepared for analysis by sectioning paraffin blocks at a thickness of five micrometers. Following the protocol outlined by Bancroft et al. (2013) for routine examination, the resultant sections were stained with hematoxylin and eosin. The histopathological quantitative score was evaluated based on the methodology described by Majeed et al. (2019). Testis tissue was evaluated using the image analysis system Leica QWin DW3000 (LEICA Imaging Systems Ltd., Cambridge, England) for the seminiferous tubular diameter, lumen diameter and epithelial thickness. For each section in each group, the six fields with the highest representation were evaluated under 100× magnification using light microscopy that was transferred into a screen.

### Estimation of DNA fragmentation using comet assay

The extent of DNA strand breaks in the testicular tissues of three distinct groups was assessed utilizing the alkaline comet assay, which was previously characterized by Tice et al. (2000). The analysis of comets was conducted using an Axio fluorescence microscope manufactured by Carl Zeiss in Germany. The microscope was supplied with an excitation filter set at 524-nm wavelength and a barrier filter set at 605 nm. The Komet 5.0 analysis system designed by Kinetic Imaging, Ltd. in Liverpool, UK, was used with a charge-coupled device (CCD) camera to quantify the percentage of migrated DNA, tail length, tail moment, and olive tail moment.

### Statistical analysis

Data were analyzed using statistical software package (IBM-SPSS) version 23 software. Kolmogorov–Smirnov test was executed to illustrate the population distribution. The normally distributed data were analyzed using one-way ANOVA test. Descriptive statistical data is displayed as mean ± standard deviation (SD). Values of *p*< 0.05 were considered significant. Cohen’s *d* effect size was calculated using GPower software version 3.1.9.4. Effect size *d* was interpreted as follows: <0.20 is a weak effect, 0.21–0.50 is a modest effect, 0.50–0.8 is a moderate, and >0.8 is a strong effect.

## Results

### Body weight and relative reproductive organs weight

In Table 1, the body weight and relative testis and epididymis weights of all groups are presented.

**Table 1:** Body weight (wt.) and relative organ weights of all the studied groups. Data is presented as mean ± standard deviation (SD)

Parameter/group	Control	THIA-treated	
		22.5 mg/kg	62.1 mg/kg
Body wt. (g)	82.40 ± 5.98	64.50 ± 5.34*	42.40 ± 11.33*#
P1, effect size	----	0.000, 3.15	0.000, 4.41
P2, effect size	----	----	0.000, 2.49
Relative right testis wt. (g)	0.98 ± 0.09	0.85 ± 0.10*	0.74 ± 0.09*#
P1, effect size	----	0.004, 1.37	0.000, 2.67
P2, effect size	----	----	0.022, 1.16
Relative left testis wt. (g)	0.98 ± 0.09	0.85 ± 0.10*	0.72 ± 0.09*#
P1, effect size	----	0.006, 1.37	0.000, 2.89
P2, effect size	----	----	0.006, 1.37
Relative right epididymis wt. (g)	0.12 ± 0.02	0.12 ± 0.04	0.12 ± 0.03
P1, effect size	----	0.634, 0.00	0.474, 0.00
P2, effect size	----	----	0.809, 0.00
Relative left epididymis wt. (g)	0.12 ± 0.02	0.11 ± 0.03	0.11 ± 0.03
P1, effect size	----	0.339, 0.39	0.163, 0.39
P2, effect size	----	----	0.649, 0.00

^*^Represent significant difference (P1<0.05), as compared to the control group

^#^Represent significant difference (P2<0.05), as compared to the THIA-treated group (22.5 mg/kg)

Effect size *d* was interpreted as follows: <0.20 is a weak effect, 0.21–0.50 is a modest effect, 0.50–0.8 is a moderate, and >0.8 is a strong effect

In comparison to the control group, rats treated with low dose (22.5 mg/kg) and high dose (62.1 mg/kg) of THIA showed significant (*p*=0.000) decline in the total body weight with a large effect size with Cohen’s *d* of 3.15 and 4.41, respectively. As compared to the rats administered 22.5 mg/kg of THIA, rats administered high dose of THIA revealed a remarkable reduction in the body weight with a large effect size of 2.49.

As compared to the control group, rats treated with low dose (22.5 mg/kg) and high dose (62.1 mg/kg) of THIA exhibited marked reductions in the relative weights of right (*p*=0.004 and *p*=0.000) and left (*p*=0.006 and *p*=0.000) testis. Thiacloprid treatment resulted in a large effect size on relative testicular weight with (*d*=1.37 and 2.67) of the right and (*d*= 1.37 and 2.89) of the left testis. Compared to the rats administered 22.5 mg/kg of THIA revealed marked declines in the relative right (*p*=0.022) and left (*p*=0.006) testis weights with (*d* =1.16 and 1.37, respectively) indicating a large effect.

Rats treated with low (22.5 mg/kg) and high (62.1 mg/kg) doses of THIA showed no change in the relative weights of the right (*p*=0.634 and *p*=0.474) and left (*p*=0.339 and *p*=0.163) epididymis; there was no effect on relative weight of epididymis with Cohen’s *d* of 0.00.

### Semen analysis outcome

#### Sperm count, motility, and viability

The sperm count as well as percentages of motility, viability and abnormality are reported (Table 2). As compared to the control group, by increasing the dose of THIA, the sperm count, motility and viability percent were significantly (*p*=0.000) reduced. THIA exposure for 8 weeks revealed a strong effect size (*d* > 0.8) on semen quality with a Cohen’s *d* of (6.57, 8.82), (20.14, 23.54), and (2.81, 9.10) for count, motility, and viability respectively. In comparison to the rats treated with low dose of THIA, the sperm count, motility and viability percent in the rats administered high dose of THIA were significantly declined with effect sizes of 3.77, 2.50, and 4.00, respectively, indicating a large effect.

**Table 2:** The sperm count, motility and viability of all the studied groups. Data is presented as mean ± standard deviation (SD)

Parameter/group	Control	THIA-treated	
		22.5 mg/kg	62.1 mg/kg
Count (×10^6^/ml)	77.33 ± 6.51	42.33 ± 3.79*	25.00 ± 5.29*#
P1, effect size	----	0.000, 6.57	0.000, 8.82
P2, effect size	----	----	0.007, 3.77
Motility (%)	44.67 ± 0.58	15.00 ± 2.00*	10.00 ± 2.00*#
P1, effect size	----	0.000, 20.14	0.000, 23.54
P2, effect size	----	----	0.010, 2.50
Viability (%)	70.67 ± 4.51	55.67 ± 6.03*	37.00 ± 2.65*#
P1, effect size	----	0.000, 2.81	0.000, 9.10
P2, effect size	----	----	0.003, 4.00

*Represent significant difference (P1<0.05), as compared to the control group

^#^Represent significant difference (P2<0.05), as compared to the THIA-treated group (22.5 mg/kg)

Effect size *d* was interpreted as follows: <0.20 is a weak effect, 0.21–0.50 is a modest effect, 0.50–0.8 is a moderate, and >0.8 is a strong effect

#### Sperm morphology

**Fig. 1:**
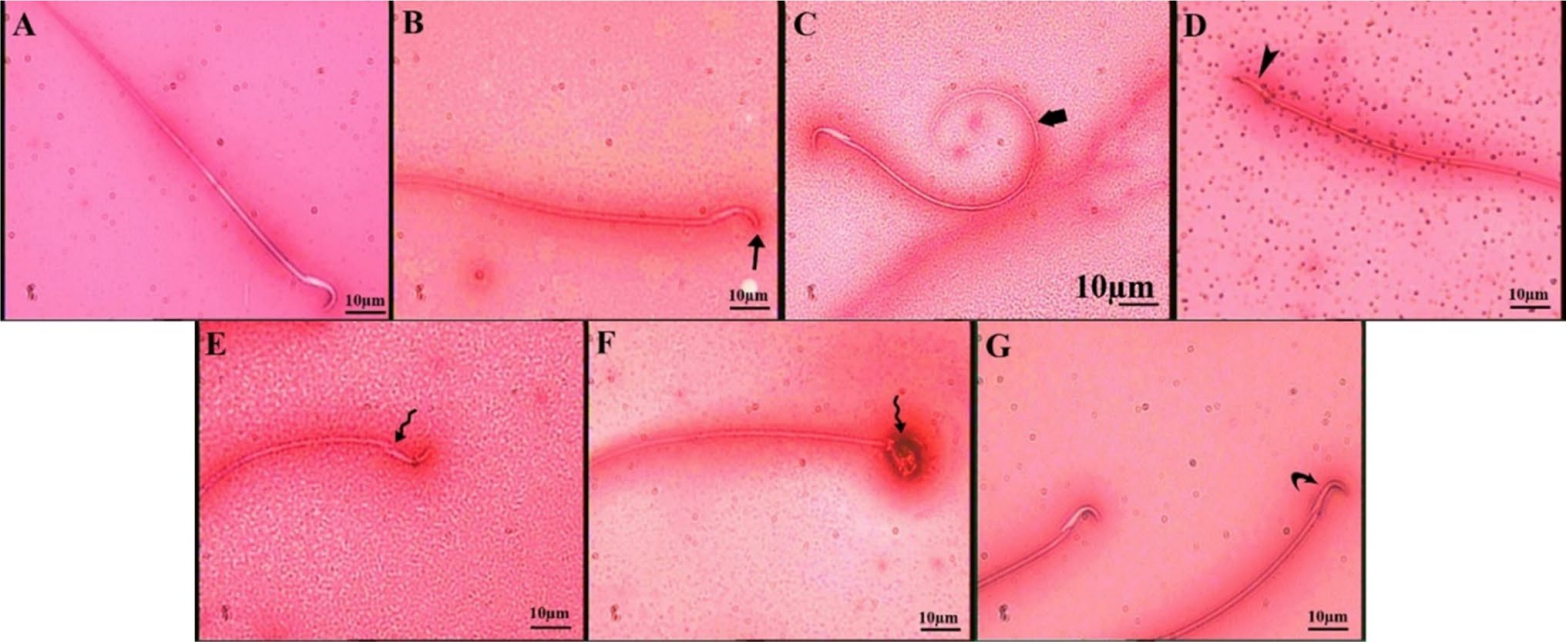
Microphotographs illustrating sperm morphologically. **A** Normal sperm (**B**–**G**) various sperm defects. (**B**, **C**: low dose) **B** Sperm with head without hook (arrow); **C** Sperm with bent tail (thick arrow); (**D**–**G**: high dose) **D** banana head (arrowhead); **E**, **F** amorphous head (wavy arrow), **G** dead head (curved arrow)

The changes in morphological abnormalities of spermatozoa such as hookless, amorphous, banana head, or abnormal tail were observed (Table 3 and Fig. 1). The percentage of sperm abnormality in the rats treated with low and high doses were significantly (*p*=0.000) greater than the control group. Sperm abnormality was remarkably (*p*=0.001) increased after administration of 62.1 mg/kg of THIA, compared to treatment with 22.5mg/kg of THIA. The effect sizes of low and high doses, as measured by Cohen’s *d*, were *d*=7.42 and 31.46 respectively, which refers to a strong effect.

**Table 3:** The percentages of morphological abnormalities in spermatozoa of all the studied groups. Data is presented as mean ± standard deviation (SD)

Parameter/group	Control	THIA-treated	
		22.5 mg/kg	62.1 mg/kg
Abnormality (%)	13.67 ± 0.58	32.33 ± 3.51*	42.33 ± 1.15*#
P1, effect size	----	0.000, 7.42	0.000, 31.46
P2, effect size	----	----	0.001, 3.83
Hookless (%)	3.33 ± 0.58	6.33 ± 1.53*	10.00 ± 2.00*#
P1, effect size	----	0.049, 2.59	0.002, 4.53
P2, effect size	----	----	0.024, 2.06
Amorphous (%)	3.00 ± 1.00	8.00 ± 1.00*	9.00 ± 1.00*
P1, effect size	----	0.001, 5.00	0.000, 6.00
P2, effect size	----	----	0.267, 1.00
Banana (%)	0.33 ± 0.58	1.33 ± 1.52	2.00 ± 1.00
P1, effect size	----	0.310, 0.87	0.114, 2.04
P2, effect size	----	----	0.488, 0.52
Abnormal tail (%)	7.00 ± 1.00	16.33 ± 1.15*	21.00 ± 2.00*#
P1, effect size	----	0.000, 8.65	0.000, 8.85
P2, effect size	----	----	0.008, 2.86

^*^Represent significant difference (P1<0.05), as compared to the control group

^#^Represent significant difference (P2<0.05), as compared to the THIA-treated group (22.5 mg/kg)

Effect size *d* was interpreted as follows: <0.20 is a weak effect, 0.21–0.50 is a modest effect, 0.50–0.8 is a moderate, and >0.8 is a strong effect

In the rats treated with 22.5 mg/kg and 62.1 mg/kg of THIA, significant elevations in the percentages of hookless, amorphous as well as the abnormal tail of sperms were detected, as compared to the control group. The rats administered high dose of THIA showed marked elevations in the percentage of hookless head (*p*=0.024) and abnormal tail (*p*=0.008) of spermatozoa, as compared to those treated with a low dose.

Effect sizes of low and high doses of THIA on the percentages of hookless, amorphous as well as the abnormal tail of sperms were (2.59, 4.53), (5.00, 6.00), and (8.65, 8.85) respectively, which indicates a large effect.

### Reproductive hormone levels

The serum levels of testosterone, FSH, and LH of all the experimental groups are presented (Table 4). In comparison to the corresponding control groups, the levels of testosterone were remarkably reduced in the rats treated with low (*p*=0.010) and high doses (p=0.000) of THIA. The effect size was large with (*d*=2.71) in 22.5 mg/kg THIA and (*d*=7.06) in the 62.1 mg/kg THIA group. As compared to the low dose treated group, a remarkable decline in the levels of testosterone (*p*=0.002) was observed with a large effect size of 5.24.

**Table 4:** Effect of thiacloprid on reproductive hormones and testicular enzymes (ng/mg protein) of all the studied groups. Data is presented as mean ± standard deviation (SD)

Parameter/group	Control	THIA-treated	
		22.5 mg/kg	62.1 mg/kg
Testosterone	10.07 ± 1.40	6.77 ± 1.00*	2.18 ± 0.73*#
P1, effect size	----	0.010, 2.71	0.000, 7.06
P2, effect size	----	----	0.002, 5.24
Follicle-stimulating hormone (FSH)	0.48 ± 0.12	0.96 ± 0.29*	1.47 ± 0.18*#
P1, effect size	----	0.032, 2.16	0.001, 6.47
P2, effect size	----	----	0.026, 2.11
Luteinizing hormone (LH)	1.43 ± 0.15	2.60 ± 0.10*	3.20 ± 0.30*#
P1, effect size	----	0.000, 9.18	0.000, 7.46
P2, effect size	----	----	0.011, 2.68
3β-hydroxysteroid dehydrogenase (3βHSD)	188.73 ± 3.23	169.40 ± 7.86*	100.67 ± 14.30*#
P1, effect size	----	0.049, 3.22	0.000, 8.49
P2, effect size	----	----	0.000, 5.96
17β-hydroxysteroid dehydrogenase (17βHSD)	5.20 ± 0.66	3.13 ± 0.67*	1.47 ± 0.25*#
P1, effect size	----	0.004, 3.11	0.000, 7.47
P2, effect size	----	----	0.011, 3.28

^*^Represent significant difference (P1<0.05), as compared to the control group

^#^Represent significant difference (P2<0.05), as compared to the THIA-treated group (22.5 mg/kg)

Effect size *d* was interpreted as follows: <0.20 is a weak effect, 0.21–0.50 is a modest effect, 0.50–0.8 is a moderate, and >0.8 is a strong effect

However, in the rats treated with low and high doses of THIA, significant elevation in the levels of FSH (*p*=0.032 and *p*=0.001) and LH (*p*=0.000 and *p*=0.000) were detected, respectively, as compared to the control group. A strong effect (*d*> 0.8) was revealed in THIA-treated groups with effect sizes of (2.16 and 6.47) of FSH level and (9.18 and 7.46) of LH level. The rats treated with high dose of THIA showed marked elevation in the levels of FSH (*p*=0.026) and LH (*p*=0.011), as compared to those administered low dose with a large effect size of 2.11 and 2.68.

### Testicular steroidogenic enzymes

The levels of the 3β-hydroxy steroid dehydrogenase (3βHSD) and 17β-hydroxy steroid dehydrogenase (17βHSD) were displayed in Table 4. Rats administered 22.5mg/kg of THIA exhibited significant declines in the activity of 3βHSD (*p*=0.049) and 17βHSD (*p*=0.004), as compared to the control group with Cohen’ *d* of 3.22 and 3.11, respectively indicating a large effect. In comparison to the control and low dose-treated groups, rats treated with high dose of THIA exhibited marked reductions in the activities of 3βHSD (*p*=0.000 and *p*=0.000) and 17βHSD (*p*=0.000 and *p*=0.011), respectively.

### Oxidative stress biomarkers

The levels of MDA, GSH as well as the activities of CAT and SOD are presented in Table 5. In comparison to the control group, rats treated with low and high doses of THIA exhibited significant elevations in the levels of MDA with a large effect size of 7.60 and 13.00, respectively. In the rats treated with high dose of THIA, the levels of MDA were significantly (*p*=0.000) greater than those treated with low dose of THIA, with a strong effect size of 7.28.

**Table 5:** The levels of malondialdehyde (MDA), glutathione (GSH) and activities of catalase (CAT) and superoxide dismutase (SOD) of all the studied groups. Data is presented as mean ± standard deviation (SD)

Parameter/group	Control	THIA-treated	
		22.5 mg/kg	62.1 mg/kg
MDA (nmol/g)	0.79 ± 0.01	0.96 ± 0.03*	1.26 ± 0.05*#
P1, effect size	----	0.001, 7.60	0.000, 13.00
P2, effect size	----	----	0.000, 7.28
GSH (mg/g)	4.06 ± 0.67	2.09 ± 0.24*	0.99 ± 0.34*#
P1, effect size	----	0.000, 3.41	0.000, 5.03
P2, effect size	----	----	0.001, 3.28
CAT (U/g)	6.40 ± 0.68	1.99 ± 0.30*	0.63 ± 0.07*#
P1, effect size	----	0.000, 8.39	0.000, 10.96
P2, effect size	----	----	0.000, 5.66
SOD (U/g)	117.72 ± 5.84	96.95 ± 7.21*	60.40 ± 11.59*#
P1, effect size	----	0.001, 2.70	0.000, 5.64
P2, effect size	----	----	0.000, 3.39

^*^Represent significant difference (P1<0.05), as compared to the control group

^#^Represent significant difference (P2<0.05), as compared to the THIA-treated group (22.5 mg/kg)

Effect size *d* was interpreted as follows: <0.20 is a weak effect, 0.21–0.50 is a modest effect, 0.50–0.8 is a moderate, and >0.8 is a strong effect

Rats administered either low or high doses of THIA exhibited marked reductions in the levels of GSH (*p*=0.000 and *p*=0.000) as well as the activities of CAT (*p*=0.000 and *p*=0.000) and SOD (*p*=0.001 and *p*=0.000), as compared to the control group. As compared to the low dose-treated group, rats administered a high dose of THIA exhibited significant reductions in the GSH content (*p*=0.001), CAT activity (*p*=0.000), and SOD activity (*p*=0.000) with large effect sizes of 3.28, 5.66, and 3.39, respectively.

### Testicular histopathological findings

**Fig. 2:**
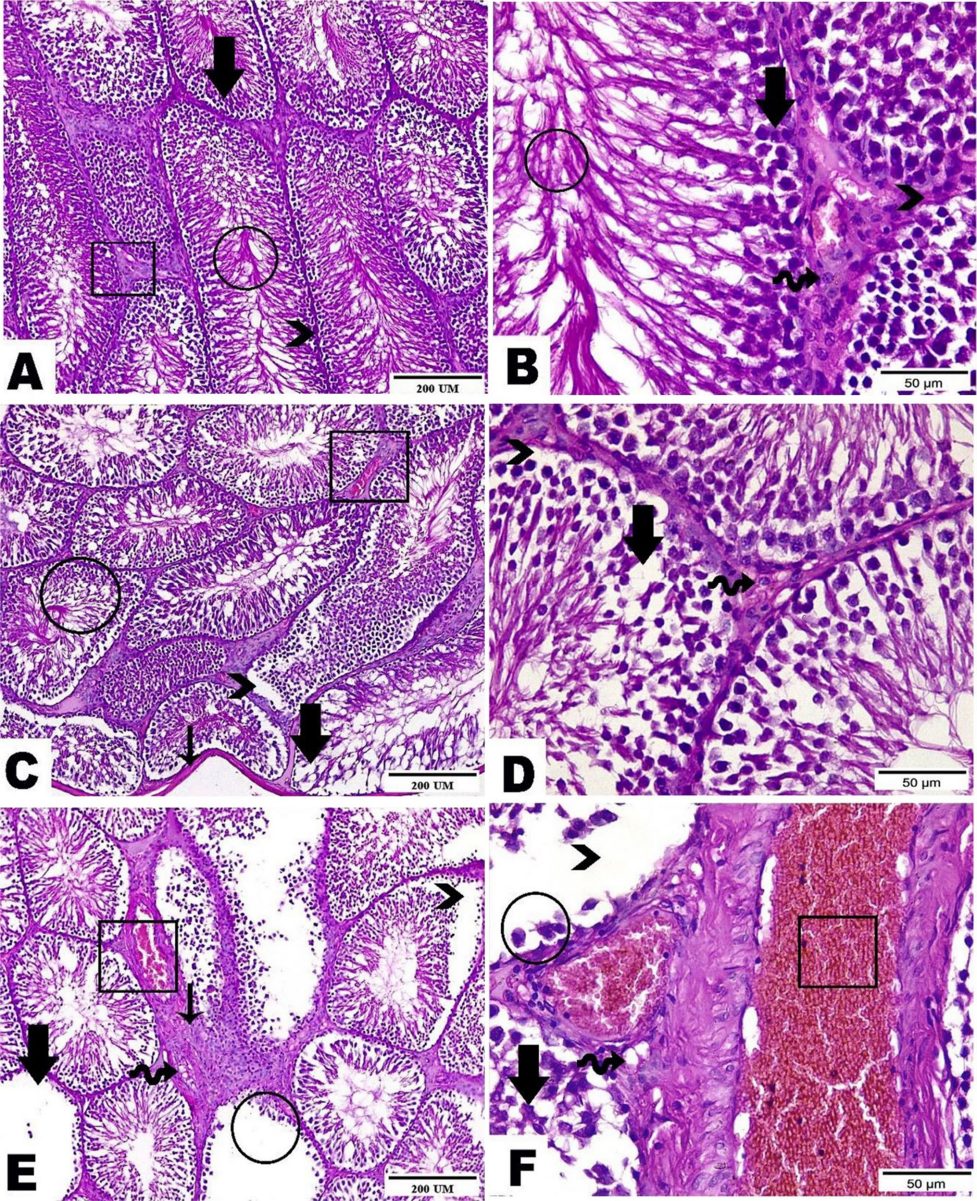
Photomicrographs presented testicular tissues stained with hematoxylin and eosin stain of the three experimental groups as follows: **A**, **B** The control group demonstrated the normal structure of seminiferous tubules and spermatogenic cells (thick arrow), sperms in its lumen (circle), basement membrane (arrowhead), normal interstitial tissue (cube) and Leydig cells (wave arrow). **C**, **D** The low dose treated group (22.5mg/kg THIA) showed few seminiferous tubules with spermatogenic cells (circle) as well as Leydig cells (wave arrow), thickening of the tunica albuginea of few seminiferous tubules (thin arrow), spermatogenic cells separated from the basement membrane (arrowhead), vacuolations in between spermatogenic cells (thick arrow), congested blood vessels in interstitial tissue (cube). **E**, **F** The high dose treated group (62.1mg/kg THIA) highlighted severe degenerative changes along seminiferous tubules and the lining of apoptotic spermatogenic cells (thick arrows). An obvious decrease in epithelial height (circle) and separation of spermatogenic cells from the basement membrane (arrowhead). Interstitial tissue marked a high accumulation of fibrous connective tissue (thin arrow), congested blood vessels (cube), and vacuolations in between Leydig cells (wave arrow). (Magnification power=×100, ×400, and scale bar=200μm, 50μm)

The testis of control rats demonstrated normal well-organized seminiferous tubules with regular basement membranes lined with successive layers of the germinal epithelium with normal supporting Sertoli cells with a lumen occupied with spermatozoa. These tubules are delineated by typical interstitial tissue housing Leydig cells (Fig. 2A, B). Testicular tissues of the low dose (22.5 mg/kg) thiacloprid-treated group revealed few pathological alterations in seminiferous tubules such as thickening of tunica albuginea, separation of spermatogenic cells from the basement membrane, vacuoles in between spermatogenic cells, along with congested blood vessels in interstitial tissue (Fig. 2C, D). In group treated with dose 62.1 mg/kg, testicular tissue revealed severe degenerative changes along seminiferous tubules such as apoptotic spermatogenic cells, decrease in epithelial height, and isolated spermatogenic cells from the basement membrane. Interstitial tissue marked high accumulation of fibrous connective tissue, blood vessels congestion, and intracellular vacuoles of Leydig cells (Fig. 2E, F).

Morphometric analysis of all experimental groups was presented in Table 6. As compared to the control group, rats treated with low dose (22.5mg/kg) and high dose (62.1mg/kg) of THIA exhibited marked reductions in the tubular diameter (*p*=0.000 and *p*=0.000) and epithelial thickness (*p*=0.000 and *p*=0.000) with effect sizes of 2.39, 5.31, 4.14, and 9.34, respectively, considered as a large effect (*d*>0.8); while in the lumen diameter, there was an unmarked reduction in the low dose and marked reduction in the high dose as compared to the control (*p*=0.473 and *p*=0.003) with an effect size of 0.42 and 2.00, respectively. As compared to the rats administered 22.5mg/kg of THIA revealed marked declines in the tubular diameter (*p*=0.003), epithelial thickness (*p*=0.000), and lumen diameter (*p*=0.013) with effect sizes of 1.88, 6.33, and 1.65, respectively indicating a large effect.

**Table 6:** The morphometric analysis of all the studied groups. Data is presented as mean ± standard deviation (SD)

Parameter/group	Control	THIA-treated	
		22.5 mg/kg	62.1 mg/kg
Tubular diameter (μm)	275.04 ± 6.40	255.97 ± 9.22*	241.05 ± 6.39*#
P1, effect size	----	0.000, 2.39	0.000, 5.31
P2, effect size	----	----	0.003, 1.88
Epithelial height (μm)	181.16 ± 6.45	157.87 ± 4.67*	126.91 ± 5.09*#
P1, effect size	----	0.000, 4.14	0.000, 9.34
P2, effect size	----	----	0.000, 6.33
Lumen diameter (μm)	93.88 ± 10.36	98.10 ± 9.58	114.15 ± 9.81*#
P1, effect size	----	0.473, 0.42	0.003, 2.00
P2, effect size	----	----	0.013, 1.65

^*^Represent significant difference (P1<0.05), as compared to the control group

^#^Represent significant difference (P2<0.05), as compared to the THIA-treated group (22.5 mg/kg)

Effect size *d* was interpreted as follows: <0.20 is a weak effect, 0.21–0.50 is a modest effect, 0.50–0.8 is a moderate, and >0.8 is a strong effect

### Comet assay of testicular tissue

**Fig. 3:**
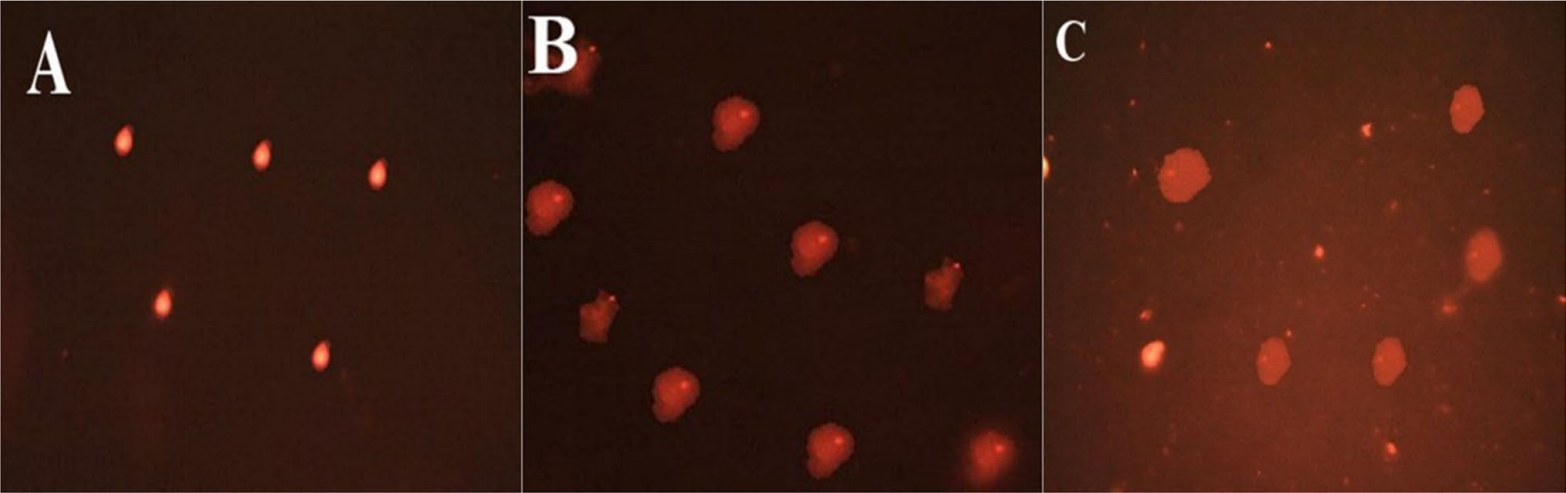
Photomicrographs of DNA damage observed through comet assay in testicular tissues. **A** Control group, **B** low dose (22.5 mg/kg) thiacloprid group, **C** high dose (62.1 mg/kg) thiacloprid group

The results of comet assay parameters are displayed (Table 7 and Fig. 3). In comparison to the control group, rats treated with low dose of THIA showed significant elevations in the tailed percentage (*p*=0.000), tail length (*p*=0.031), DNA damage in tail (*p*=0.000), and tail moment (*p*=0.032) with effect sizes of 3.14, 1.25, 2.22, and 1.58, respectively, which referrers to large effect.

**Table 7:** The comet assay parameters of all the studied groups. Data is presented as mean ± standard deviation (SD)

Parameter/group	Control	THIA-treated	
		22.5 mg/kg	62.1 mg/kg
Tailed (%)	10.78 ± 1.13	14.37 ± 1.16*	22.80 ± 1.64*#
P1, effect size	----	0.000, 3.14	0.000, 8.54
P2, effect size	----	----	0.000, 5.93
Tail length (px)	8.18 ± 0.81	10.51 ± 2.51*	13.59 ± 2.55*#
P1, effect size	----	0.031, 1.25	0.000, 2.86
P2, effect size	----	----	0.005, 1.22
Tail DNA (%)	3.54 ± 1.05	6.79 ± 1.78*	6.11 ± 1.98*
P1, effect size	----	0.000, 2.22	0.003, 1.62
P2, effect size	----	----	0.389, 0.36
Tail moment	0.25 ± 0.16	0.65 ± 0.32*	0.88 ± 0.53*
P1, effect size	----	0.032, 1.58	0.001, 1.61
P2, effect size	----	----	0.187, 0.53
Olive tail moment	0.56 ± 0.05	0.92 ± 0.16	1.30 ± 0.64*#
P1, effect size	----	0.056, 3.03	0.000, 1.63
P2, effect size	----	----	0.046, 0.81

*Represent significant difference (P1<0.05), as compared to the control group

^#^Represent significant difference (P2<0.05), as compared to the THIA-treated group (22.5 mg/kg)

Effect size *d* was interpreted as follows: <0.20 is a weak effect, 0.21–0.50 is a modest effect, 0.50–0.8 is a moderate, and >0.8 is a strong effect

Moreover, rats treated with high dose of THIA exhibited marked elevations in tailed percentage (*p*=0.000), tail length (*p*=0.000), DNA damage in tail (*p*=0.003), tail moment (*p*=0.001), and olive tail moment (*p*=0.000), in comparison to the control group. The effect size was large with a Cohen’s *d* of 8.54, 2.86, 1.61, and 1.63 respectively.

As compared to the rats treated with low dose of THIA, rats treated with 62.1 mg/kg THIA showed remarkable elevations in the tailed percent, tail length, and olive tail moment, with large effect sizes of 5.93, 1.22, and 0.81, respectively. A modest effect was observed in DNA damage with an effect size of 0.36.

## Discussion

Considering recent reports of thiacloprid toxicity to non-target species and humans, long-term evaluations of its mammalian reproductive toxicity are needed. This eight-week study examined the toxicity of thiacloprid to the male Wistar rats’ reproductive system.

After eight weeks of treatment, thiacloprid lowered adult Wistar rats’ weight change compared to the control group. Comparable effects were shown in male and female rabbits exposed to different THIA doses for 45 days and in developing mice exposed to 100 mg/kg THIA for 4 weeks (Islam 2022; Zou et al. 2023). The reduction in weight gain could be ascribed to decreased food consumption and insecticide-induced gastrointestinal tract malabsorption (Saber et al. 2021).

The obtained data showed that THIA-treated groups had significantly decreased relative testis weights than the control group. This reduction might be attributed to the testosterone deficiency necessary for male reproductive organ growth and function (Saber et al. 2021). However, recent study on acetamiprid, sulfoxaflor and thiacloprid reproductive toxicity in rats and immature mice reported no significant change in testicular weight (Arıcan et al. 2020; Mohamed et al. 2022; Zou et al. 2023). The current study observed no change in the relative epididymal weight of the treated groups. In contrast, imidacloprid decreased epididymal weights in developing and adult male rats (Bal et al. 2012a, b; Saber et al. 2021).

Thiacloprid is an endocrine disruptor (Caron-Beaudoin et al. 2017) that acts through disrupting the antioxidant defense system (Wang et al. 2018) and induces oxidative stress by ROS production (Kammoun et al. 2019). It can create free radicals either directly or via P450 cytochrome throughout its metabolism. Superoxide radicals are converted into hydroxyl radicals via hydrogen peroxide (Weidinger and Kozlov 2015). In the current study, thiacloprid significantly increased MDA levels, especially at higher dosages, and decreased CAT, GSH, and SOD activity in testicular tissue. Aydin (2011) reported that acute and subacute thiacloprid administration increased lipid peroxidation and lowered the levels of catalase (CAT) and glutathione peroxidase (GPx) in the spleen, thymus, and bone marrow, while SOD activity lowered in the thymus and raised in the bone marrow and spleen. Recently, thiacloprid has shown the ability to cause oxidative stress in the testicular tissue of rats (Kammoun et al. 2017). Similarly, previous studies demonstrated that oxidative stress is the mechanism by which imidacloprid causes reproductive damage by reporting increased lipid peroxidation and decreased CAT, SOD, GSH, and GPx levels in male rats (Bal et al. 2012a; Yang and Lee 2015; Lonare et al. 2016; Mahajan et al. 2018; Saber et al. 2021).

The main type of oxidative stress is attributed to the formation of free radical and lipid peroxidation may harm spermatozoa membrane lipid structure causing motility loss, membrane integrity degradation, and sperm dysfunction (Adedara et al. 2018). In the present study, thiacloprid decreased sperm count, motility, viability, and increased morphological abnormalities in rats compared to control group. Zou et al. (2023) confirmed that 100 mg/kg THIA for 4 weeks decreased spermatogenesis-related gene mRNA and increased sperm abnormalities. Similar toxic effects comprised lower sperm viability, motility, concentration, and abnormal morphology have been reported in neonicotinoid studies (Arıcan et al. 2020; Saber et al. 2021; Mohamed et al. 2022).

Unbalanced reactive oxygen species and antioxidant defense systems can disturb the hypothalamic-pituitary-gonadal axis, which controls spermatogenesis, causing male infertility (Darbandi et al. 2018). The present study found that THIA reduced serum testosterone and increased LH and FSH in rats. Recent rabbit and rat studies revealed thiacloprid-induced testosterone deficiency, which may impair reproductive functions (Islam 2022; Zou et al. 2023). Male rats subjected to neonicotinoids acetamiprid, imidacloprid, and sulfoxaflor for 90, 56, and 28 days showed similar effects (Arıcan et al. 2020; Saber et al. 2021; Mohamed et al. 2022). Decreased levels of testosterone may induce negative feedback from the hypothalamus-pituitary-testicular (HPT) axis, raising serum LH and FSH (Kong et al. 2017; Sai et al. 2014).

Oxidative stress in the testis destroys steroidogenesis and spermatogenesis (Chainy et al. 2009). 3ꞵ-hydroxysteroid dehydrogenase (3ꞵ-HSD) and 17ꞵ-hydroxysteroid dehydrogenase (17ꞵ-HSD) are essential enzymes for converting dehydroepiandrosterone (DHEA) into testosterone (Payne and Hales 2004). In the present study, thiacloprid-treated groups exhibited a significant reduction in the levels of the 3β-3βHSD and 17βHSD than those measured in the control group. In line with our results, the activity of 3ꞵ-HSD and 17ꞵ-HSD was significantly lowered in rats treated with imidacloprid and acetamiprid (Lonare et al. 2014; Mosbah et al. 2017). Habotta et al. (2021) and Saber et al. (2021) revealed notable disruptions in testosterone biosynthesis pathways and gene expression in response to imidacloprid and thiamethoxam neonicotinoids in male rats. Thiacloprid was also found to inhibit testosterone biosynthesis by decreasing STAR and CYP11A1 content (Zou et al. 2023).

The oxidative damage caused by the generation of different oxidants from cellular proteins, lipids, and DNA can lead to DNA damage (Ismail and Mohamed 2012). Insecticides have been linked to several forms of DNA damage, as base-free sites, base modifications, deletions, frame shifts, and chromosomal rearrangements, all of which are caused by the insecticides' artificial production of ROS (Naziroğlu et al. 2009). Our results showed significant DNA damage in the two treated groups compared to control as indicated by all comet parameters. Kammoun et al. (2017) reported extensive DNA damage in the testicular tissue of thiacloprid treated rats. In previous studies, thiacloprid exposure caused DNA oxidative damage in bovine peripheral lymphocytes, whole blood cultures, human peripheral lymphocytes, rat bone marrow, and chicken embryos (Calderón-Segura et al. 2012; Kocaman et al. 2014; Schwarzbacherová et al. 2019; Galdíková et al. 2019; Farag et al. 2021). The study of Verebová et al. (2019) summarized that thiacloprid modified the stability and structure of calf thymus DNA by binding into the minor groove via hydrogen or hydrophobic interactions.

These alterations were confirmed microscopically by the presence of degeneration changes of seminiferous tubules with apoptotic distribution of spermatogenic cells. DNA fragmentation was also seen in rat testicular tissue after imidacloprid exposure in immature and mature rats (Mohamed et al. 2017). Zang et al. (2000) and Bal et al. (2012b) found that 90 days of treatment with imidacloprid (2 and 8 mg/kg) caused apoptosis and DNA fragmentation of rat semen.

Upon examining the testicular histopathology, the present study found that the two doses of thiacloprid induced several alterations in the seminiferous tubules and Leydig cells. Previous studies revealed that testicular damage caused by oxidative stress (Tetsatsi et al. 2019; Habotta et al. 2021). Similar testicular histopathological alterations were previously found in thiacloprid-treated rats (Kammoun et al. 2017) and rabbits (Islam 2022).

## Conclusion

The obtained results contribute to the elucidation of thiacloprid impairs the reproductive functions of Wistar male rats. Reduced sperm quality, testicular tissue degeneration, disturbance of hormones and testicular enzymes, oxidative stress, and DNA damage are the main causes of adverse consequences. This research highlights the need to adopt safeguards against insecticide exposure in the occupational and the environment. Further studies are needed to clarify the effect of thiacloprid residues in the environment on the spermatogenic and steroidogenic parameters and how it impairs the reproductive health of humans.

## Data Availability

Data supporting this study are included within the article.
